# Chemical Composition of Essential Oils and Local Knowledge of *Myrica gale* in Lithuania

**DOI:** 10.3390/plants12051050

**Published:** 2023-02-26

**Authors:** Kristina Ložienė, Viktorija Maskolaitytė, Juozas Labokas, Jurga Būdienė, Vaida Vaičiulytė

**Affiliations:** 1 Institute of Botany, Nature Research Centre, Žaliųjų Ežerų g. 47, LT-12200 Vilnius, Lithuania; 2 Pharmacy Center, Institute of Biomedical Sciences, Faculty of Medicine, Vilnius University, M. K. Čiurlionio g. 21/27, LT-03101 Vilnius, Lithuania; 3 Institute of Ecology, Nature Research Centre, Akademijos g. 2, LT-08412 Vilnius, Lithuania; 4 Center for Physical Sciences and Technology, Department of Organic Chemistry, Saulėtekio al. 3, LT-10257 Vilnius, Lithuania

**Keywords:** fruits, habitat, leaves, monoterpenes, respondent, sesquiterpenes, survey

## Abstract

*Myrica gale* L. (Myricaceae) is an essential oil-bearing plant that is rare in Lithuania and naturally grows only in the western part of the country. The aim of this study was to analyze the composition of essential oils of *Myrica gale* in different habitats in Lithuania and in different parts of the plant, as well as evaluate the local knowledge about *M. gale* as a medicinal and aromatic plant. Samples of fruits and leaves (from one and three *M. gale* populations, respectively) were studied separately. Essential oils were isolated from dried fruits and leaves by hydrodistillation and analyzed by GC/FID and GC/MS methods. Results showed that *M. gale* fruits accumulated 4.03 ± 2.13% essential oils, meanwhile leaves—up to 19 times less. A total of 85 compounds were identified in the essential oils of the *M. gale*. Monoterpene hydrocarbons accounted for about half of the total essential oil content; meanwhile, either monoterpene hydrocarbons or sesquiterpene hydrocarbons (depending on habitat) dominated in leaves. The main compounds (depending on habitat) in essential oils of fruits and leaves were α-pinene, 1,8-cineole, limonene, *δ*-cadinene, and (*E*)-nerolidol. The high variation in the composition of *M. gale* essential oils suggests the presence of different chemotypes within the studied habitats of this plant. Evaluation of local knowledge of *M. gale* through the survey of 74 residents of 15 villages in western Lithuania showed that only 7% of respondents knew this plant. Poor knowledge of *M. gale* could be related to the narrow range of the natural species’ distribution in Lithuania.

## 1. Introduction

It was estimated that worldwide production of essential oils increased from 45,000 to more than 150,000 tons a year over the period of 1990–2017; a similar trend was observed in the European Union, where production of essential oils increased from about 29,000 to 41,000 tons a year over the period of 2006–2016. Moreover, several economic analyses showed that the growth would continue [1]. The same source reports that the demand for essential oils comes from the following major markets: food and beverages (35%), fragrances, cosmetics and aromatherapy (29%), households (16%), and pharmaceuticals (15%). In general, medicinal and aromatic plant products are getting more and more popular for their natural bioactive compounds in many developed countries. Of the two basic approaches to satisfy the ever-growing needs for plant raw material, natural collection from wild populations is preferred by customers over field cultivation, particularly those using essential oils as aroma [2]. This finding suggests that natural essential oil-bearing plants are highly valued because of their quality and thus continue to be of interest to researchers and natural product developers. A small proportion of essential oil-bearing plant species occur in such northern latitudes as the Baltic Sea region, of which Lithuania is a part of when compared to, e.g., the Mediterranean or any other region with a similar climate. Nevertheless, the populations of some interesting species of that category can be found there too; one such species is the sweet gale.

The sweet gale or bog myrtle (*Myrica gale* L., Myricaceae Rich. ex Kunth.), a small deciduous shrub, is distributed across the northern hemisphere, including northern and western Europe, North America, and east Asia, preferring oceanic climates [3]. Studies report that the essential oils of *Myrica gale* L. exhibit antimicrobial [4,5,6], antiviral [4], insecticidal or repellent [7,8,9], antifungal [10,11], and anticancer [12,13] activities. The medicinal uses of the different plant parts of *M. gale* include those related to diuretic, styptic, anticathartic, and anthelmintic effects [14,15,16]. Although a variety of different compounds and their combinations in *M. gale* are biologically active, their volatile compounds, or essential oils in general, play one of the most important roles in the applications of this plant material. The essential oils accumulate in most parts of the plant, including the leaves, branches, catkins, and fruits. However, their quantitative and qualitative composition may differ significantly depending not only on the plant part but also on the environmental conditions, such as climate and habitat type, as well as plant population and seasonal variations [17]. An important biological property of *M. gale* is that it is a subdioecious species, i.e., having most, but not all, individuals that are dioecious. As observed in Wales, UK, the predominantly or strictly male stems were about twenty times as frequent as the predominantly or strictly female stems [18]. Similar observations were reported from Poland [19], but with the ratio between male and female shoots as 3:1, respectively. In any case, this fact adds some variability in terms of essential oil accumulation in individual plants, as female plants are distinguished by their essential oil-rich fruit, as shown in our recent study [5].

Due to their strong preference for an oceanic climate and a lack of suitable habitats, *M. gale* is a rare species in Lithuania and occurs naturally only in the Tyrai and Svencelė fens (Kliošiai Landscape Reserve and Svencelė Telmological Reserve, respectively) in the coastal zone of the country [20]. According to the literature, *M. gale* still occurred in the vicinity of Šventoji (Kretinga district) [21], but now it is no longer found there due to wetland drainage and peat exploitation works. In the publications of Lithuanian authors, it is indicated that *M. gale* was used to repel moths at homes, in the production of beer and wine, and as an antibacterial agent [22,23,24,25]. Therefore, we found it appropriate to investigate whether the local inhabitants of this part of Lithuania know *M. gale* today and whether their knowledge of its use is not fading out along with the shrinking occurrence of the species itself.

The aim of this study was to analyze and compare the composition of the essential oils of *M. gale* in different habitats and in different plant parts, as well as evaluate the local knowledge about *M. gale* as a medicinal and aromatic plant.

## 2. Results

### 2.1. Percentage Distribution of Leaves, Fruits, and Twigs in the Raw Material of the Myrica gale

The established mass percentages of the air-dried twigs, leaves, and fruits showed that the leaves made up more than half of the total raw material—58.8 ± 8.3%. Meanwhile, the percentages of twigs (without leaves and fruits) and fruits were 31.0 ± 8.8% and 10.2 ± 4.4%, respectively.

### 2.2. Quantitative Composition of Essential Oils in the Leaves and Fruits of the Myrica gale

The mean percentages of essential oil in the leaves were quite similar across all studied habitats and varied from 0.21–0.27% (*v*/*w*) (Table 1). However, as seen from the coefficients of variation, the individual plant variations in the Svencelė (CV = 38%) and Kliošiai (CV = 48%) habitats were more than twice as high as those from Verkiai (CV = 18%).

As seen in Table 1, the mean percentage of *M. gale* fruit essential oil content in the Svencelė habitat was more than 19 times higher than that of the leaves. However, the plants from the Svencelė habitat showed high variation in fruit essential oil accumulation; the highest percentage amounted to 9.11% which exceeded the lowest one by nearly six times (CV = 53%). Spearman’s correlation test did not show any statistically significant correlation between the percentages of essential oils in the leaves and fruits of *M. gale* individuals.

### 2.3. Chemical Composition of Essential Oils in Leaves and Fruits of Myrica gale

**Figure 1:**
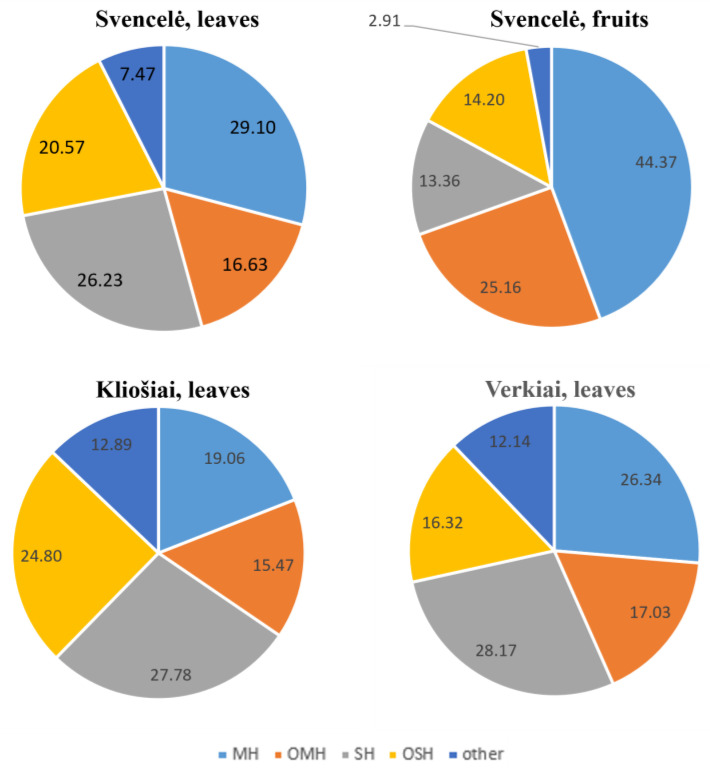
Percentage distribution of the terpene classes in the essential oils of the leaves and fruits of *Myrica gale* growing in different habitats (MH—monoterpene hydrocarbons, OM—oxygenated monoterpenes, SH—sesquiterpene hydrocarbons, and OS—oxygenated sesquiterpenes).

Eighty-five different chemical compounds were identified, which represented 84.19–98.69% and 92.12–99.67% of the total essential oil amount in *M. gale* leaves and fruits, respectively. Most of these compounds belong to four classes of terpenes: monoterpene hydrocarbons, oxygenated monoterpenes, sesquiterpene hydrocarbons, and oxygenated sesquiterpenes (14, 20, 27, and 15 chemical compounds, respectively) (Table 2, Figure 1).

Monoterpene hydrocarbons comprised the main group of compounds in the fruits of *M. gale* and accounted for about half of the total essential oil content; meanwhile, two groups of compounds, depending on habitat, dominated more or less in the leaves—either monoterpene hydrocarbons or sesquiterpene hydrocarbons (Figure 1, Table 2). Monoterpene hydrocarbons and sesquiterpene hydrocarbons in the leaves and fruits varied between individuals within wider ranges if compared with oxygenated monoterpenes and oxygenated sesquiterpenes, respectively (Table 2). Percentages of monoterpene hydrocarbons negatively correlated with those of sesquiterpene hydrocarbons in the leaves of *M. gale* in Svencelė, Kliošiai and Verkiai (r = −0.68, r = −0.62, and r = −0.90, respectively) and in the fruits of *M. gale* in Svencelė (r = −0.53); however, these correlations were statistically significant (*p* < 0.05) only in the leaves from Svencelė and Verkiai.

The main compounds of monoterpene hydrocarbons fraction present both in the leaves and fruits of *M. gale* were α-pinene and limonene (Table 2). The most abundant compound in fruit essential oils was α-pinene; its percentage was twice higher in fruits than in leaves in the Svencelė habitat, and the t-test showed a significant (*p* < 0.05) difference in its content between the two parts of the plant studied. Spearman’s correlation analysis showed a positive but not statistically significant correlation between the contents of α-pinene in the essential oils of *M. gale* leaves and fruits. Percentages of limonene were very similar in both the leaves and fruits. Also, a high percentage of fruit essential oil was accounted for by the monoterpene hydrocarbon α-phelandrene in the Svencelė habitat; its percentage in fruit essential oils was on average three times higher and, in contrast to α-pinene, statistically significantly (*p* < 0.05) and differed from that percentage in the leaves.

The most abundant compound, 1,8-cineole, was in the fraction of oxygenated monoterpenes, and its percentage in fruit essential oils was on average twice as high as that of leaves. Spearman’s correlation analysis showed a statistically significant (*p* < 0.05) positive correlation (r = 0.66) between percentages of eucalyptol in the essential oils of the leaves and fruits of *M. gale* growing in the Svencelė habitat.

Among the most abundant compounds of the sesquiterpene fraction in the essential oils of leaves and fruits in all habitats were δ-cadinene, (E)-nerolidol and germacrone and accounted for an average of 5–13%, except for the germacrone in the leaves of *M. gale* growing in the Verkiai habitat, where the average of this compound was less than half a percent in essential oil.

### 2.4. Evaluation of Local Knowledge about the Myrica gale

**Figure 2:**
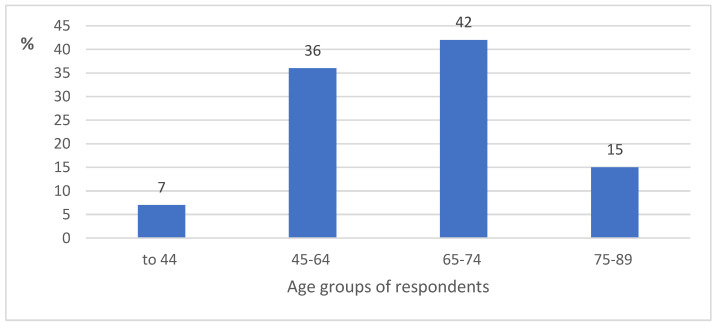
Percentage distribution of the respondents (N = 74) by their age groups.

In total, 74 respondents took part in the survey for the evaluation of local knowledge about the species. The respondents’ distribution by age is as follows: up to 44 years old—5; 45–64 years old—27; 65–74 years old—31; and 75–89 years old—11 respondents. The percentage distribution of the respondents by age groups is presented in Figure 2. More than three-quarters of all respondents were women. The survey revealed that only 7% of all respondents (two women from Svencelė, one woman and one man from Kintai, and one man from Rusnė) knew of *M. gale*. One respondent told us that as a child he was taught to identify *M. gale* by his grandmother, while the other respondents knew this plant from books and/or booklets of the Svencelė Telmological Reserve, radio broadcasts, etc. Almost all of them were middle-aged, except for the man from Rusnė, who represented the elderly group. Spearman’s correlation analysis showed a strong (r = 0.71) but not statistically significant correlation between the respondents’ time of familiarity with *M. gale* and their age. The respondents who lived closer to the Svencelė habitat knew *M. gale* for a shorter time (only 5–8 years), while those who lived in more remote places from this habitat (as in Kintai and Rusnė) knew this plant for a longer time. The distance of the respondents’ place of residence from the Svencelė habitat of *M. gale* had no significant influence on the identification of the species.

## 3. Discussion

Our previous study showed that the fruits of *M. gale* accumulated 3.34 ±0.05% and 2.71 ± 0.22% of essential oils in the natural population of Kliošiai, western Lithuania, and in the anthropogenic population of Verkiai, eastern Lithuania, respectively [5], which is significantly less than that obtained by our current study in the Svencelė population (4.03 ± 2.13%). A high variation between individuals (CV = 53%) in the latter population may suggest the presence of different chemotypes of *M. gale* in the Svencelė population. Moreover becasue the species is propagated not only by their rhizomes but also by their seed [20]. So far, no leaf essential oil analysis has been carried out in the Lithuanian populations of *M. gale*. Therefore, a comparison of our data on leaf essential oil (0.21–0.27%, Table 1) is only possible with foreign data. Thus, the literature showed that essential oils in *M. gale* leaves were significantly lower and amounted to 0.11–0.13% in Scotland and Finland [28], and 0.13–0.16% in Poland [6]. Meanwhile, content was significantly higher in France, amounting to 0.38% [11]. This variation could be related to different climatic conditions as well as plant-material collecting time. It should be noted that the raw materials of *M. gale* are usually harvested by cutting stems and branches. Thus, it is useful to know the mass percentage of leaves on branches, as they provide most of the essential oils and may contribute to a better estimation of the overall essential oil content. In our study, the leaves made up more than half of the total dried raw material—58.8%. A very close percentage of leaf biomass was established in Finland (61.5%) and considerably higher—in Scotland (73.3%) [28]. However, these percentages may vary significantly depending on harvesting time, which in turn is related to plant development stage, predetermining the quantity and quality of the essential oils. Our study suggests that harvesting branches with mature fruits from female plants may provide the highest total yields of *M. gale* essential oils.

In regard to the terpene groups, the monoterpenes (a total 69.53% of all identified compounds) clearly dominated in the fruit essential oils of the *M. gale*, while sesquiterpenes were prevalent in the leaves, particularly in the Kliošiai habitat (a total of 52.58%) (Figure 1). Our previous study [5], showed even higher percentages of fruit monoterpenes (74.16% in Verkiai and 76.69% in Kliošiai). Data in the literature also showed that the fruits of *M. gale* accumulate more monoterpenes than the leaves. For example, in Estonia, *M. gale* fruits contained 19–41% monoterpene hydrocarbons and 34–44% oxygenated monoterpenes, while sesquiterpene hydrocarbons and oxygenated sesquiterpenes amounted to 7–14% and 3–9%, respectively [29]. In Canada, the leaves of *M. gale* accumulated way more sesquiterpenes (53.10%) than monoterpenes (38.02%) [30].

The monoterpene, α-pinene, was the most abundant in both leaf and fruit essential oils; however, fruit essential oil was on average more than twice as rich with this compound as leaf essential oil (amounting to 21.34%). Our earlier study also showed that α-pinene was prevalent in the fruit essential oil of *M. gale* in the Kliošiai and Verkiai habitats (amounting to 27.17% and 23.52%, respectively) [5]. Literature sources provide a variety of data in this regard: in Japan, α-pinene only amounted up to 1.9% in the leaf essential oil of the *M. gale*; in Canada—2.2%; in France—12.2%; in Finland—17.4%; however, in Scotland it reached even up to 32.2% [13,28,30,31]. Interestingly, Finnish, Scottish, and French populations of *M. gale* are distinguished by higher amounts of α-pinene in the leaves than in the fruits [30,31]. α-Pinene and some other monoterpenes, like limonene and citronellol, are recognized as having high antimicrobial and repellent activity [32,33] which may be the reason why *M. gale* has been used as a repellent. In addition, α-pinene chemotype (as well as *β*-elemenone and 1,8-cineole ones) of *M. gale* were reported as having antibacterial activity [34]. Meanwhile, the other isomer of pinene, *β*-pinene, amounted to an eight times lower percentage than that of α-pinene in the essential oil of both parts of *M. gale* plants and was not detected in some individuals at all.

The second most abundant compound was 1,8-cineole which was way more abundant in fruit essential oils than those of the leaves. Although its maximum content in some individuals amounted to 16.38% (leaves, Kliošiai) and 24.01% (fruits, Svencelė), there were individuals within the same populations without 1,8-cineole. Additionally, in terms of compound distribution among individuals, the same pattern was observed with α-pinene. This finding may suggest the occurrence of respective chemotypes, however, this case is regarding the qualitative composition of EO. Literature sources report different amounts of 1,8-cineole accumulating in the fruit essential oil of *M. gale* with the closest percentages established in Finland (14.7%) and France (18.9%) [11,28].

In addition to α-pinene and 1,8-cineole, the fruits and leaves of *M. gale* were rich in monoterpene limonene and sesquiterpenes *δ*-cadinene and (*E*)-nerolidol with average amounts comparable to each other in all habitats (Table 2). In the Verkiai habitat, *M. gale* individuals were distinguished by very low average amounts of germacrone and the lowest individual maximum value in leaf EO. This finding suggests that the Verkiai population may contain a chemotype with low or absent germacrone content in leaf EO. The amounts of the other above mentioned major compounds (1,8-cineole, α-pinene, limonene, *δ*-cadinene and (*E*)-nerolidol) varied within very wide limits even in the same habitats. This finding implies that the composition of essential oil may differ significantly even within the same relatively small habitats indicating that chemical polymorphism could be common for the Lithuanian populations of *M. gale* too as it was reported by Krogsbøll et al. [34] on chemotypes (α-pinene, *β*-elemenone, (*E*)-nerolidol, and 1,8-cineol type) comprising 24 Scottish genotypes of *M. gale* grown in Denmark. However, to obtain a better picture of the chemotypic variation of *M. gale* in essential oils in Lithuania, the populations should be investigated more thoroughly, e.g., by employing cultivation trials of selected clones. The more so, the studied populations proved themselves as potential sources for the selection of certain chemotypes.

New opportunities appear with the advancement of the techniques used for essential oil extraction. The most recent research carried out with the Irish *M. gale* [35] found that quantitative differences in essential oil content occurred between extraction methodologies: microwave-assisted hydrodistillation yielded higher quantities of monoterpene and sesquiterpene hydrocarbons, while Clevenger hydrodistillation targeted their oxygenated counterparts.

The study of the local knowledge on *M. gale* in 15 villages located near the Svencelė Telmological Reserve demonstrated that only 7% of all respondents correctly identified the *M. gale*. Most of the knowledge about the species was not inherited locally but rather based on different outside sources, predominantly literature. A wide array of folkloric and traditional applications of the *M. gale*—from aromatizer and spice to pharmaceutical, dye, and insect repellent—described in literature sources [15,16,17,36,37] in contrast to the scarce knowledge of the species among the local people suggest certain reasons why. First, the former generation with the knowledge has already passed away along with the narrowing range of the natural species distribution (an occurrence of the species was formerly recorded in the northern coastal area of the country that is no longer there [20]). Second, the species was too scarce in Lithuania to get any deeper folkloric recognition. *M. gale* has been included in the national list of protected species since 1962, and most recently, the red list evaluation assigned the species to the IUCN category Vulnerable (VU) [20]. This assignment prevents the collection and use of *M. gale* from local populations. However, cultivation of introduced *M. gale* genotypes remains an option.

## 4. Materials and Methods

### 4.1. Sampling of Myrica gale Raw Material

Samples of raw material of *M. gale* were collected from two natural habitats in western Lithuania (the Kliošiai Landscape Reserve and Svencelė Telmological Reserve, both in the Klaipėda District Municipality) and from an anthropogenic population in eastern Lithuania (the Verkiai Regional Park, Vilnius City Municipality). The voucher specimens of *M. gale* have been deposited in the Herbarium of Nature Research Centre (BILAS, Vilnius, Lithuania); the numbers of the specimens are 92609 (Kliošiai), 55064 (Svencelė), and 92610 (Verkiai). All habitats are mesotrophic fens. Twigs with fruits and leaves were collected from 10, 10, and 5 female individuals separately from the Kliošiai, Svencelė and Verkiai habitats, respectively, in August and dried at an ambient room temperature, protected from direct sunlight. Dried plant raw material was kept in paper bags. Leaves and fruits were separated from twigs before the essential oil isolation.

### 4.2. Isolation and Analysis of Essential Oils

Essential oils were isolated from all dried samples of *M. gale* fruits and leaves separately by hydrodistillation in Clevenger type apparatus for two hours (European Pharmacopoeia Commission 2008); unground fruits were used for the isolation of essential oils. The distillation of essential oils from each sample was carried out in three replications and stored in the freezer until further analysis.

The essential oil solutions of 1% were prepared in the mixture of diethyl ether and n-pentane (1:1) for further investigations. The identification of compounds of essential oils was carried out by employing a Shimadzu GC-2010 coupled with Shimadzu GCMS-QP 2010 plus mass selective detector (Shimadzu, Japan). Separation of compounds was performed on a fused silica (100% dimethyl polysiloxane) column (30 m × 0.25 mm ID × 0.25 µm film thickness, Restek, USA), splitless injection; helium as carrier gas at a flow rate of 1.6 mL/min, injector and detector temperatures 250 °C. The GC oven temperature was programmed as follows: initially temperature 50 °C (isothermal for 7 min) increased to 250 °C at the rate 4 °C/min (isothermal for 5 min) and further increased at the rate of 30 °C/min to 300 °C, the final temperature was kept for 2 min. Mass spectra in electron mode were generated at 70 eV. The identification of the compounds was based on the comparison of computer mass spectra library (NBS75K) with the probability not less than 90%, retention indices (RIs) [26,27] and analytical standards of α-pinene, 1,8-cineole, β-caryophylene, β-pinene, p-cymene, myrcene, caryophyllene oxide, terpinen-4-ol, borneol, γ-terpinene, limonene, α-terpinene, and camphene were purchased from Sigma-Aldrich (Germany). The retention indices were determined relative to the retention times of a series of n-alkanes (C_7_–C_30_) with linear interpolation. The quantitative analyses of the main compounds were carried out using a FOCUS GC (Thermo Scientific) gas chromatograph with flame ionisation detector (FID) on the silica capillary column TR-5MS (30 m × 0.25 mm ID × 0.25 μm film thickness) (Thermo Electron Corporation, USA) under the same chromatographic conditions. The percentages of the investigated compounds were recalculated according to the areas of the FID chromatographic peaks assuming that all constituents of the essential oil comprise 100%.

### 4.3. Evaluation of Local Knowledge about Myrica gale

**Figure 3:**
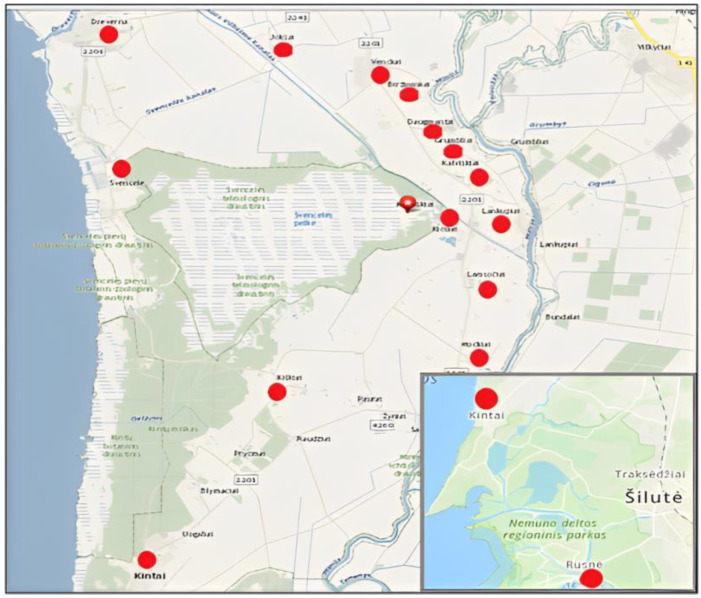
Map of *Myrica gale* survey area in Western Lithuania. Red circles indicate locations of inter-views taken from residents.

The survey to evaluate local knowledge about *M. gale* was caried out in 15 villages located around the Svencelė Telmological Reserve: Svencelė, Kiošiai, Kalviškiai, Daugmantai, Grumbliai, Lamsočiai, Mockai, Biržininkai, Jokšai, Venckai, Kiškiai, Lankupiai, Kintai, Rusnė, and Dreverna (see map, Figure 3). Respondents were grouped into four age groups based on the provisional guidelines for international standard age classifications [38]. The age groups were as follows: young people up to 44 years old, middle-aged people from 45 to 64 years old, elderly people from 65 to 74 years old, and people from 75 up to 89 years. A structured survey was applied with the questionnaire containing 15 questions. During the survey a live twig of *M. gale* was demonstrated to the interviewees to learn whether they could identify the species. Each respondent was interviewed for 5–30 min depending on how much knowledge they had about the plant.

### 4.4. Statistical Analysis

Spearman‘s correlation analysis and t-tests were carried out with STATISTICA^®^ 10. The significance level α = 0.05 was chosen, and the results were considered statistically significant when the *p*-value was less than 0.05. Microsoft Excel 2016 was used for data tabulation and processing as well as chart making.

## 5. Conclusions

*M. gale* fruits accumulate significantly higher amounts of essential oils (up to 19 times) if compared to the leaves, suggesting that harvesting of *M. gale* branches or twigs with fruits could be the more effective way to maximize essential oil yields. This factor also should be considered when establishing plantations of *M. gale* crops for essential oil production, as this species is largely dioecious. The option of cultivating this species should not be ignored, particularly in the countries where *M. gale* is evaluated as a threatened or near threatened species, as in Lithuania. A total of 85 compounds were identified in *M. gale* essential oils, most of which belong to monoterpenes and sesquiterpenes. The fruit essential oils are distinguished by their chemical composition from those of the leaves in that they accumulate monoterpene hydrocarbons in prevailing quantities and account for about half of the total essential oil content. Meanwhile, two groups of compounds, depending on habitat, dominated in the leaves—either monoterpene hydrocarbons or sesquiterpene hydrocarbons. Based on the high variation in both quantitative and qualitative composition of *M. gale* essential oils, one could judge the presence of different chemotypes within the studied populations of *M. gale*. This finding indicates that the Lithuanian populations of *M. gale* are potential sources for the selection of target genotypes of the species. However, in order to further prove this finding, some more detailed research is needed.

The questionnaire-based survey revealed a scarce knowledge of the species among local people, obtained from secondary sources such as books and media rather than inherited from their ancestors due to the loss of knowledge during the natural change of generations and the rarity of the species itself.

## Figures and Tables

**Table 1:** Percentages of essential oil in the leaves and fruits of the *Myrica gale* growing in different habitats. CV—coefficient of variation.

Habitat	Mean ± SD	Min–Max
	Leaves (%)	
Svencelė (N = 10)	0.21 ± 0.08	0.10–0.33
Kliošiai (N = 10)	0.27 ± 0.13	0.09–0.50
Verkiai (N = 5)	0.22 ± 0.04	0.18–0.28
	Fruits (%)	
Svencelė (N = 10)	4.03 ± 2.13	1.57–9.11

**Table 2:** Chemical composition of the essential oils in the leaves and fruits of *Myrica gale* growing in different habitats.

Compound (Chemical Group)	RI [26,27]	Leaves						Fruits	
		Svencelė (N = 10)		Kliošiai (N = 10)		Verkiai (N = 5)		Svencelė (N = 10)	
		Mean ± SD	Min–Max	Mean ± SD	Min–Max	Mean ± SD	Min–Max	Mean ± SD	Min–Max
*α*-Thujene (MH)	930	0.16 ± 0.00	nd–0.16	0.09 ± 0.06	nd–0.13	0.06 ± 0.01	nd–0.06	0.28 ± 0.11	nd–0.39
*α*-Pinene (MH)	939	10.32 ± 5.97	2.26–19.29	5.47 ± 3.15	nd–10.08	6.78 ± 3.75	2.94–11.45	21.34 ± 9.77	nd–33.2
Camphene (MH)	954	0.49 ± 0.16	nd–0.70	0.24 ± 0.07	nd–0.32	0.23 ± 0.14	0.09–0.41	0.91 ± 0.22	nd–1.23
Benzaldehyde	960	1.33 ± 2.26	nd–4.71	nd	nd	nd	nd	nd	nd
Octen-3-ol-1	963	nd	nd	0.56 ± 0.31	nd–1.22	0.60 ± 0.21	0.38–0.85	nd	nd
*β*-Pinene (MH)	979	1.24 ± 0.67	0.32–2.49	nd	nd	nd	nd	2.79 ± 0.41	nd–3.48
Myrcene (MH)	990	1.35 ± 0.65	0.53–2.22	3.00 ± 3.34	nd–11.52	1.34 ± 0.48	0.78–2.09	2.02 ± 0.70	nd–2.87
*α*-Phelandrene (MH)	1002	3.90 ± 2.21	1.1–7.47	1.41 ± 1.19	nd–4.19	1.46 ± 0.29	1.20–1.92	9.99 ± 3.80	nd–15.88
*α*-Terpinene (MH)	1018	0.69 ± 0.20	nd–1.08	0.35 ± 0.26	nd–0.65	0.25 ± 0.05	0.19–0.32	1.43 ± 0.47	nd–1.93
*p*-Cymene (MH)	1024	1.96 ± 0.83	0.43–2.98	2.61 ± 1.20	nd–4.35	3.25 ± 0.79	2.20–4.05	0.94 ± 0.28	nd–1.2
Limonene (MH)	1029	5.63 ± 2.55	1.17–9.11	5.94 ± 2.75	nd–10.27	6.87 ± 1.53	4.78–8.98	6.45 ± 2.12	nd–9.47
*β*-Phellandrene (MH)	1030	2.37 ± 1.18	nd–3.41	nd	nd	nd	nd	2.01 ± 0.00	nd–2.01
*1,8*-Cineole (OM)	1031	9.87 ± 3.71	3.34–14.36	7.56 ± 5.87	nd–16.38	9.28 ± 3.32	6.66–14.79	16.40 ± 5.83	nd–24.01
(*Z*)-*β*-Ocimene (MH)	1037	nd	nd	2.22 ± 1.87	nd–4.47	3.23 ± 1.24	nd–4.42	nd	nd
(*E*)-*β*-Ocimene (MH)	1050	0.66 ± 0.16	nd–0.84	0.48 ± 0.38	nd–1.00	0.86 ± 0.43	0.41–1.39	0.34 ± 0.11	nd–0.44
*γ*-Terpinene (MH)	1054	1.95 ± 0.70	0.47–2.72	1.49 ± 1.03	nd–3.23	2.09 ± 0.54	1.67–2.79	2.48 ± 0.62	nd–3.30
Lavandulol tetrahydro	1157	nd	nd	1.16 ± 1.18	nd–4.28	0.32 ± 0.11	0.19–0.47	nd	nd
Acetophenone	1065	0.28 ± 0.10	nd–0.35	nd	nd	nd	nd	0.33 ± 0.17	nd–0.57
Terpinolene (MH)	1088	0.55 ± 0.08	nd–0.68	nd	nd	nd	nd	0.79 ± 0.11	nd–0.94
Linalool (OM)	1096	0.28 ± 0.04	nd–0.34	0.51 ± 1.15	nd–0.69	0.42 ± 0.12	0.33–0.63	0.39 ± 0.15	nd–0.64
Nonanal	1101	0.37 ± 0.06	nd–0.48	nd	nd	nd	nd	nd	nd
Fenchol (OM)	1116	0.17 ± 0.00	nd–0.17	nd	nd	nd	nd	0.18 ± 0.04	nd–0.21
(*Z*)-*p*-Menth-2-en-1-ol (OM)	1121	0.12 ± 0.00	nd–0.12	nd	nd	nd	nd	0.30 ± 0.10	nd–0.48
Alloocimene (OM)	1132	nd	nd	0.09 ± 0.01	nd–0.10	0.04 ± 0.00	nd–0.04	0.20 ± 0.06	nd–0.29
Borneol (OM)	1169	0.41 ± 0.43	nd–1.28	0.30 ± 0.12	nd–0.49	0.28 ± 0.08	0.22–0.39	0.27 ± 0.14	nd–0.50
Terpinen-4-ol (OM)	1177	1.41 ± 0.66	0.52–2.90	1.29 ± 0.76	0.32–2.32	1.36 ± 0.32	1.05–1.71	2.19 ± 0.89	1.10–4.29
*α*-Terpineol (OM)	1188	1.90 ± 1.11	0.75–4.69	1.73 ± 0.91	0.24–2.76	1.92 ± 0.38	1.47–2.36	2.33 ± 1.21	1.08–4.54
(*E*)-Sabinol (OM)	1190	nd	nd	nd	nd	nd	nd	0.15 ± 0.04	nd–0.20
(*Z*)-Piperitol (OM)	1196	nd	nd	nd	nd	nd	nd	0.17 ± 0.06	nd–0.23
Citronellol (OM)	1225	0.85 ± 0.64	nd–1.79	nd	nd	nd	nd	0.37 ± 0.14	nd–0.46
Benzylacetone	1226	0.78 ± 1.13	nd–3.56	nd	nd	nd	nd	nd	nd
Nerol (OM)	1229	nd	nd	0.76 ± 2.41	nd–7.62	0.73 ± 0.80	nd–1.29	nd	nd
Neral	1238	nd	nd	0.56 ± 1.78	nd–5.62	0.44 ± 0.49	nd–0.78	nd	nd
Geraniol (OM)	1252	nd	nd	0.13 ± 3.95	nd–12.49	0.98 ± 1.29	nd–2.46	0.32 ± 0.23	nd–0.48
Geranial	1267	nd	nd	1.39 ± 1.28	nd–3.75	0.67 ± 0.47	0.42–1.51	nd	nd
Bornyl acetate (OM)	1288	0.53 ± 0.11	nd–0.67	0.41 ± 0.17	nd–0.74	0.34 ± 0.06	0.27–0.40	0.78 ± 0.24	0.56–1.30
Methyl acetate	1290	nd	nd	0.88 ± 0.95	nd–3.2	0.52 ± 0.36	0.23–1.15	nd	nd
Carvacrol (OM)	1300	nd	nd	0.69 ± 2.19	nd–6.91	0.64 ± 0.74	nd–1.49	nd	nd
*p*-Mentha-1,4-dien-7-ol (OM)	1332	0.74 ± 0.35	nd–1.1	nd	nd	nd	nd	0.67 ± 0.54	nd–1.63
*α*-Cubebene (SH)	1348	nd	nd	2.09 ± 1.40	nd–3.90	1.71 ± 0.98	0.20–2.89	nd	nd
α-Terpinyl acetate (OM)	1349	0.79 ± 0.60	nd–1.84	0.78 ± 0.66	nd–2.16	0.89 ± 0.023	0.65–1.25	1.35 ± 0.83	nd–2.28
Citronellyl acetate (OM)	1350	1.28 ± 0.66	nd–2.67	0.15 ± 0.00	nd–0.15	0.11 ± 0.00	nd–0.11	2.67 ± 2.65	nd–8.50
Neryl acetate (OMH)	1361	nd	nd	1.01 ± 0.76	nd–1.87	1.21 ± 1.40	nd–2.81	nd	nd
*α*-Copaene (SH)	1375	2.03 ± 1.19	nd–3.14	0.06 ± 0.00	nd–0.06	0.17 ± 0.11	nd–0.29	1.09 ± 0.44	nd–1.90
*β*-Bourbonene (SH)	1388	nd	nd	0.53 ± 0.00	nd–0.53	0.04 ± 0.00	nd–0.04	nd	nd
*α*-Gurjunene (SH)	1402	0.81 ± 0.20	nd–1.07	nd	nd	nd	nd	0.36 ± 0.16	nd–0.64
(*E*)-Caryophyllene (SH)	1420	2.48 ± 1.54	nd–5.19	4.13 ± 4.97	nd–7.66	1.67 ± 0.14	1.45–1.82	1.22 ± 0.52	nd–1.90
*γ*-Elemene (SH)	1430	0.59 ± 0.23	nd–0.80	nd	nd	nd	nd	nd	nd
*β*-Copaene (SH)	1432	0.29 ± 0.07	nd–0.39	1.13 ± 0.44	nd–1.85	1.66 ± 0.66	nd–2.37	nd	nd
Neryl acetone	1434	0.30 ± 0.08	nd–0.44	nd	nd	nd	nd	nd	nd
*α*-Humulene (SH)	1454	nd	nd	0.07 ± 0.00	nd–0.07	nd	nd	nd	nd
(*E*)-*β*-Farnesene (SH)	1456	nd	nd	0.30 ± 0.23	nd–0.46	0.12 ± 0.00	nd–0.12	nd	nd
Alloaromadendrene (SH)	1460	0.29 ± 0.07	nd–0.39	nd	nd	nd	nd	0.69 ± 0.68	nd–1.17
(*Z*)-Cadina-1,(6)-4-diene (SH)	1472	1.25 ± 0.82	nd–2.49	nd	nd	nd	nd	2.20 ± 1.28	nd–4.09
*γ*-Gurjunene (SH)	1474	nd	nd	0.18 ± 0.08	nd–0.3	0.08 ± 0.02	nd–0.10	nd	nd
(*E*)-*β*-Ionone (SH)	1488	0.36 ± 0.18	nd–0.74	0.52 ± 0.25	nd–0.82	0.34 ± 0.11	0.22–0.47	nd	nd
*β*-Selinene (SH)	1490	0.48 ± 0.21	nd–0.77	nd	nd	nd	nd	0.17 ± 0.02	nd–0.18
*σ*-Selinene (SH)	1492	nd	nd	0.68 ± 0.59	nd–1.89	0.55 ± 0.24	nd–0.75	nd	nd
(*Z*)-*β*-Guaiene (SH)	1493	0.64 ± 0.11	nd–0.72	3.10 ± 0.00	nd–3.1	0.28 ± 0.33	nd–0.66	nd	nd
*α*-Selinene (SH)	1500	0.70 ± 0.00	nd–0.70	nd	nd	nd	nd	nd	nd
Bicyclogermacrene (SH)	1500	nd	nd	0.63 ± 0.522	nd–1.54	0.68 ± 0.31	0.22–0.99	nd	nd
*α*-Muurolene (SH)	1500	0.97 ± 0.57	nd–1.98	0.91 ± 0.42	nd–1.26	1.07 ± 0.33	0.69–1.54	0.63 ± 0.48	nd–1.33
(*E,E*)-*α*-Farnesene	1502	0.64 ± 0.11	nd–0.72	nd	nd	nd	nd	nd	nd
*β*-Bisabolene (SH)	1506	nd	nd	2.22 ± 3.50	nd–7.42	0.90 ± 0.76	nd–1.98	nd	nd
*γ*-Cadinene (SH)	1523	2.27 ± 0.95	nd–4.29	3.84 ± 3.10	nd–9.12	4.59 ± 3.31	0.10–8.16	1.3 ± 1.54	nd–4.86
(*E*)-Calamenene (SH)	1522	2.02 ± 1.88	0.29–5.8	3.67 ± 2.29	0.19–7.72	3.42 ± 1.24	1.27–4.43	nd	nd
*δ*-Cadinene (SH)	1512	10.99 ± 8.32	nd–26.06	9.10 ± 6.18	0.29–18.52	7.41 ± 3.86	1.21–11.61	7.04 ± 9.91	nd–31.02
(*Z*)-Cadina-1,4-diene (SH)	1535	3.89 ± 2.53	nd–8.57	nd	nd	nd	nd	2.21 ± 3.00	nd–9.49
*α*-Cadinene (SH)	1538	2.26 ± 1.59	nd–4.81	nd	nd	nd	nd	1.27 ± 0.75	nd–2.11
Selina 3,7 (11)-diene (SH)	1545	2.02 ± 1.88	0.29–4.80	2.90 ± 3.20	0.32–9.98	4.55 ± 3.17	0.41–7.90	1.35 ± 1.05	nd–2.56
(*E*)-Nerolidol (OS)	1563	8.93 ± 6.69	1.84–18.44	13.44 ± 8.64	1.07–24.71	9.84 ± 4.99	5.11–15.74	7.00 ± 8.42	nd–26.17
Caryophyllene oxide (OS)	1583	nd	nd	0.89 ± 0.76	nd–0.98	0.29 ± 0.09	nd–0.40	nd	nd
Gleenol (OS)	1587	0.49 ± 0.36	nd–1.22	nd	nd	nd	nd	0.45 ± 0.25	nd–0.68
(*Z*)-*β*-Elemenone (OS)	1589	0.56 ± 0.30	nd–0.77	1.46 ± 1.41	nd–3.83	0.20 ± 0.05	nd–0.26	nd	nd
Ledol (OS)	1602	0.77 ± 0.56	nd–1.92	nd	nd	nd	nd	1.06 ± 1.22	nd–2.88
Humulene epoxide II (OS)	1609	0.75 ± 0.47	nd–1.58	nd	nd	nd	nd	0.68 ± 0.00	nd–0.68
1,10-di-epi-Cubenol (OS)	1619	1.53 ± 0.89	nd–2.42	nd	nd	nd	nd	1.18 ± 0.80	nd–2.23
1-epi-Cubenol (OS)	1628	4.36 ± 3.34	nd–10.9	nd	nd	nd	nd	5.07 ± 6.97	nd–19.04
Cadin-4-en-7-ol (OS)	1637	nd	nd	0.91 ± 0.66	nd–2.18	1.09 ± 0.27	nd–1.30	nd	nd
T-Muurolol + *α*- Muurolol (OS)	1640	1.67 ± 0.97	nd–3.49	4.65 ± 2.35	nd–8.75	2.73 ± 1.50	0.40–4.38	1.82 ± 2.46	nd–6.17
Selina 3,11-dien-6-*α*-ol (OS)	1642	nd	nd	0.99 ± 0.73	nd–2.44	1.42 ± 0.37	nd–1.63	nd	nd
*α*-Cadinol (OS)	1650	0.61 ± 0.35	nd–1.23	0.70 ± 0.22	nd–0.99	0.37 ± 0.30	nd–0.69	0.84 ± 1.01	nd–2.34
Aromadendrene oxide (OS)	1672	nd	nd	nd	nd	nd	nd	0.49 ± 0.45	nd–0.80
Germacrone (OS)	1693	6.63 ± 6.36	nd–18.44	8.47 ± 8.33	nd–20.37	0.40 ± 0.44	nd–0.89	5.19 ± 5.32	nd–14.75
Eudesm-7(11)-en-4-ol (OS)	1700	nd	nd	0.88 ± 0.56	nd–1.83	1.16 ± 0.80	nd–1.98	nd	nd
Monoterpene hydrocarbons (MH)		29.10 ± 14.83	6.75–47.76	19.06 ± 13.33	nd–44.02	26.34 ± 7.21	16.53–36.42	44.37 ± 22.86	nd–65.23
Oxygenated monoterpenes (OM)		16.63 ± 6.77	6.66–29.52	15.47 ± 9.41	3.28–30.56	16.83 ± 7.53	11.64–29.83	25.16 ± 8.05	9.59–32.42
Sesquiterpene hydrocarbons (SH)		26.23 ± 15.34	4.92–52.89	27.78 ± 10.34	13.7–47.45	28.17 ± 8.52	18.99–38.69	13.36 ± 16.18	2.07–53.93
Oxygenated sesquiterpenes (OS)		20.00 ± 6.66	11.91–28.93	24.80 ± 6.70	15.10–36.67	16.32 ± 4.63	10.72–21.18	13.20 ± 10.49	1.19–35.13

## Data Availability

The datasets generated for this study are available on request to the corresponding author.
